# Network pharmacology analysis reveals neuroprotective effects of the Qin-Zhi-Zhu-Dan Formula in Alzheimer’s disease

**DOI:** 10.3389/fnins.2022.943400

**Published:** 2022-10-20

**Authors:** Wenxiu Xu, Beida Ren, Zehan Zhang, Congai Chen, Tian Xu, Shuling Liu, Chongyang Ma, Xueqian Wang, Qingguo Wang, Fafeng Cheng

**Affiliations:** ^1^School of Traditional Chinese Medicine, Beijing University of Chinese Medicine, Beijing, China; ^2^Dongzhimen Hospital, Beijing University of Chinese Medicine, Beijing, China; ^3^Institute for Brain Disorders, Beijing University of Chinese Medicine, Beijing, China; ^4^School of Traditional Chinese Medicine, Capital Medical University, Beijing, China

**Keywords:** Alzheimer’s disease, Qin-Zhi-Zhu-Dan Formula, TNF signaling pathway, network pharmacology, inflammatory response

## Abstract

There is yet no effective drug for Alzheimer’s disease (AD) which is one of the world’s most common neurodegenerative diseases. The Qin-Zhi-Zhu-Dan Formula (QZZD) is derived from a widely used Chinese patent drug–Qing-Kai-Ling Injection. It consists of *Radix Scutellariae*, *Fructus Gardeniae*, and *Pulvis Fellis Suis*. Recent study showed that QZZD and its effective components played important roles in anti-inflammation, antioxidative stress and preventing brain injury. It was noted that QZZD had protective effects on the brain, but the mechanism remained unclear. This study aims to investigate the mechanism of QZZD in the treatment of AD combining network pharmacology approach with experimental validation. In the network pharmacology analysis, a total of 15 active compounds of QZZD and 135 putative targets against AD were first obtained. Gene Ontology (GO) enrichment analysis and Kyoto Encyclopedia of Genes and Genomes (KEGG) pathway analysis were then applied to clarify the biological mechanism. The anti-inflammatory mechanism of QZZD was proved, and a synthetic pathway–TNFR1-ERK1/2-NF-κBp65 signaling pathway was obtained. On the basis of the above discoveries, we further validated the protective effects QZZD on neurons with an APP/PS1 double transgenic mouse model. Weight change of the mice was monitored to assess QZZD’s influence on the digestive system; water maze experiment was used for evaluating the effects on spatial learning and memory; Western blotting and immunohistochemistry analysis were used to detect the predicted key proteins in network pharmacology analysis, including Aβ, IL-6, NF-κBp65, TNFR1, p-ERK1/2, and ERK1/2. We proved that QZZD could improve neuroinflammation and attenuate neuronal death without influencing the digestive system in APP/PS1 double transgenic mice with dementia. Combining animal pharmacodynamic experiments with network pharmacology analysis, we confirmed the importance of inflammation in pathogenesis of AD, clarified the pharmacodynamic characteristics of QZZD in treating AD, and proved its neuroprotective effects through the regulation of TNFR1-ERK1/2-NF-κBp65 signaling pathway, which might provide reference for studies on treatment of AD in the future.

## Introduction

The aging population in the world sees increasing incidence of Alzheimer’s disease (AD) (Hansen et al., 2018). It is estimated that there will be 12.7 million AD patients by 2050 (Vitali et al., 2021). Care for individuals with AD or other dementias costs about one trillion dollars a year in the US, which imposes great economic burden on the society; by 2030, this number is projected to double (Patterson, 2018). AD remains the sixth leading cause of death among individuals aged 65 and above (Kumar et al., 2022). Older adults with AD are also more vulnerable to disability and poor health (Jiang and Li, 2016). The early symptoms of AD are memory loss and learning impairment (Anger, 1991; Li et al., 2018; Kumar et al., 2022). In 1907, Dr. Alois Alzheimer first described the histopathology of AD, including brain atrophy, amyloid plaques (extracellular deposits of aggregated Aβ peptides), neurofibrillary tangles, neuronal and synaptic degeneration, and dystrophic neurites (Alzheimer, 1907). Though a great deal of manpower, resources and money have been invested in studies on this disease over the past century, there is still no effective drug for its prevention and treatment (Liang et al., 2022). As AD is a complex disease involving many factors, its etiology and pathogenesis are not clear, and single-target, single-acting drugs cannot delay the progression (Ye et al., 2022). At present, there are many studies (Yu et al., 2020; Wang X. et al., 2021) on the application of therapeutic methods of traditional Chinese medicine (TCM), such as Chinese herbal medicine and acupuncture, in the treatment of AD, which provides some enlightenment.

Traditional Chinese medicine has been used for stroke (Liu et al., 2018), asthma (Kao et al., 2018), psoriasis (Wu et al., 2018) and other complex diseases (Jiang et al., 2021; Wang Z. Y. et al., 2021) in China for over 2,000 years. Chinese herbal medicine has expanded the opportunity space for understanding physiopathology and drug development for neurodegenerative diseases, including Parkinson’s disease (Chen J. et al., 2022), multiple sclerosis (Song et al., 2017), Huntington disease (Teseo et al., 2021), AD (Luo et al., 2020) and so on. The Qin-Zhi-Zhu-Dan Formula (QZZD) is composed of *Radix Scutellariae*, *Fructus Gardeniae*, and *Pulvis Fellis Suis*. It is derived from a traditional Chinese patent drug–Qing-Kai-Ling Injection that is approved by the China Food and Drug Administration (No. Z13020880, China) (Gao et al., 2019; Ma et al., 2019), and has been clinically applied in the treatment of ischemic stroke for nearly thirty years in China (Ma et al., 2019; Zhang et al., 2020). Clinical needs and efficacy were taken into consideration for the prescription of QZZD based on our previous studies (Li et al., 2019; Li C. et al., 2021; Liu et al., 2022). We preliminarily confirmed that the main components of QZZD could alleviate brain injury and improve function of the brain by modulating inflammation, oxidative stress, apoptosis, and neurotrophic factors expression (Li et al., 2019; Li C. et al., 2021). The specific mechanism of QZZD against AD, however, was yet not fully understood.

Network pharmacology provides a new paradigm in study on traditional medicines (Zhou et al., 2020). It combines computational and experimental tools to discover a new class of therapeutic agents, understand mechanism of treatment of complex diseases, and describe the “multi-component, multi-target, and multi-pathway” characteristics of TCM (Mi et al., 2020). This approach has inspired a lot of studies on the treatment of complex diseases by TCM, such as heart disease (Tao et al., 2013; Yang et al., 2022) and AD (Luo et al., 2020).

**FIGURE 1:**
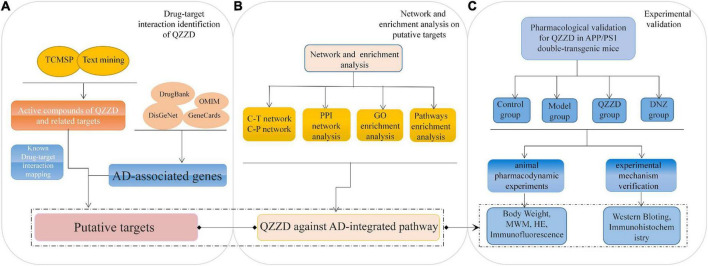
Flowchart of network pharmacology approach to mechanism of QZZD against Alzheimer’s disease. **(A)** Drug-target interaction identification of QZZD. **(B)** Network and enrichment analysis (on putative targets). **(C)** Experimental validation (*in vivo* to understand the pharmacological mechanism of QZZD against Alzheimer’s disease).

We designed a basic pharmacodynamic experiment and further combined the network pharmacology method for a comprehensive study of the pharmacodynamic mechanisms involved in the treatment of AD (Figure 1).

## Materials and methods

### Network pharmacology analysis

#### Identification of active compounds of Qin-Zhi-Zhu-Dan Formula and related targets

To find active compounds of QZZD and related targets, we utilized Traditional Chinese Medicine Systems Pharmacology Database and Analysis Platform (TCMSP)^1^ (Ru et al., 2014) in combination with text mining in Google Scholar^2^, China National Knowledge Infrastructure (CNKI)^3^ and PubMed.^4^ The calculation method of ADME/T (Absorption, Distribution, Metabolism, Excretion, and Toxicity) (Hasan et al., 2022) was selected, and we determined the specific measurement principles of ADME/T according to Lipinski’s rule (Lipinski et al., 2001; Lipinski, 2004). The rule bases pharmacokinetic properties such as absorption, distribution, metabolism and excretion (ADME) on specific molecular properties such as molecular weight under 500 Da (*MW* < *500*), partition coefficient less than 5 (*AlogP* < *5*), no more than 5 hydrogen bond donors (*Hdon* < *5*), and no more than 10 hydrogen bond acceptors (*Hacc* < *10*) (Lipinski et al., 2001; Benet et al., 2016). *AlogP* value represents the partition coefficient between octanol and water, and is of great significance for measuring hydrophobicity of molecules (Wolk et al., 2014). *Hdon* and *Hacc*, these two parameters play a role in compound-target interaction to influence absorption, distribution, metabolism, and excretion parameters (Huang et al., 2020). The active compounds of QZZD were identified according to the above principles (*MW* < *500*, *AlogP* < *5*, *Hdon* < *5*, and *Hacc* < *10*), on the basis of which, related targets were obtained after removing the duplicates.

#### Collection of Alzheimer’s disease-associated genes

Genes related to AD were collected from several public gene-related databases, including DrugBank database^5^ (Wishart et al., 2018), Online Mendelian Inheritance in Man (OMIM) database^6^ (Li et al., 2014), DisGeNET database^7^ (Piñero et al., 2021), and GeneCards database^8^ (Fishilevich et al., 2016). The integration of multiple well-recognized disease databases facilitates a comprehensive understanding of disease-related features and ensures a comprehensive and accurate collection of AD-associated genes. To rank the gene-disease association, DisGeNET database developed gene-disease association (GDA) score. It takes into account the number and type of sources (level of curation, organisms), and the number of publications supporting the association. The targets with GDA score greater than 0.1 from DisGeNET database were considered as highly correlated with AD in this study. The relevance score in GeneCards database indicates the correlation between genes and diseases. With reference to other researches (Gao et al., 2022), screening criterion for targets in GeneCards database was set as a relevance score of no less than 20. All of the targets from the OMIM and Drugbank databases were included as there were no criteria in the two databases. After removal of duplicates, AD-related targets were finally obtained. In addition, the UniProt database^9^ (Zhou et al., 2018) was used to normalize AD-related targets.

#### Acquisition of common targets between Qin-Zhi-Zhu-Dan Formula and Alzheimer’s disease

The previously obtained genes from see sections “Identification of active compounds of Qin-Zhi-Zhu-Dan Formula and related targets” and “Collection of Alzheimer’s disease-associated genes” were imported into Venny 2.1.^10^ A Venn diagram was constructed, and the common genes were considered as putative targets of QZZD against AD.

#### Construction and analysis of protein-protein interaction network

The protein-protein interaction (PPI) network (Wang et al., 2020) reflects behaviors and properties of biomolecules, which helps to understand the role of multiple proteins in complex diseases such as AD. The Search Tool for the Retrieval of Interaction Gene/Proteins (STRING) database^11^ is commonly used to construct PPI network in network pharmacology studies (Yao et al., 2022; Ye et al., 2022; Yu et al., 2022). The purpose of the STRING database is to collect, score and integrate all publicly available sources of protein-protein interaction information, and to supplement the information with computational predictions (Szklarczyk et al., 2019). The potential therapeutic target genes of QZZD were imported into the STRING database to obtain the PPI network. The data with a comprehensive score greater than 0.7 (Supplementary Table 1) were imported into Cytoscape 3.7.1 (Shannon et al., 2003) for visualization and analysis. The core target genes in the network were obtained and visualized by R software (version 4.0.3).^12^

#### Gene Ontology and Kyoto Encyclopedia of Genes and Genomes pathway enrichment analysis

The clusterProfiler package in Bioconductor (version 3.1.2) (Yu et al., 2012) and R software (version 4.0.3) were used to determine the gene IDs of the potential therapeutic targets, and then the functional enrichment analysis of gene ontology (GO) and the enrichment analysis based on Kyoto Gene and Genome Encyclopedia (KEGG) were carried out using the above two software programs. The threshold was set as *p* < 0.05, and the biological processes or pathways of the lowest *p* value were identified after excluding irrelevant pathways. GO enrichment was mainly used to understand the main action process of the target, and the KEGG pathway analysis was used to observe distribution of the targets in the pathway. Then, we chose the top 10 pathways for construction of the “Target-Pathway” network of QZZD in the treatment of AD and used Cytoscape 3.7.1 for visualization. Furthermore, in order to investigate the roles of protein targets in the pathophysiological network of AD and how QZZD acts on AD by regulating certain pathways, an “AD-integrated pathway” was proposed based on our present understanding of AD pathology and target identification (Luo et al., 2020).

### Experimental validation

#### Drugs and reagents

*Radix Scutellariae* and *Fructus Gardeniae* were purchased from Anxing Chinese Herbal Medicine Co., Ltd. (No. 200901, China). *Pulvis Fellis Suis* was purchased from Shengtai Chinese Herbal Medicine Co., Ltd. (No. 190901, China). Donepezil hydrochloride (DNZ) was obtained from Eisai China Inc. (H20070181, Shanghai, China).

#### Qin-Zhi-Zhu-Dan Formula preparation

Extracts of the three herbs in QZZD (*Radix Scutellariae*, *Fructus Gardeniae*, and *Pulvis Fellis Suis*) were mixed at a ratio of 10:6:1.5. The extraction and quality control were implemented according to our previous study (Xu et al., 2022). *Radix Scutellariae* was decocted with water 12 times of its volume twice, 1 h each time. The filtrate was concentrated to a relative density of 1.05–1.10 before adding 2 mol/L hydrochloric acid solution at 80°C to ensure that the pH value ranges from 1.0 to 2.0, which is followed by centrifugal separation and drying. *Fructus Gardeniae* was extracted with 75% ethanol as the solvent 3 times, 8 h each time. The extracts were combined and filtrated. All of the concentrated filtrates were evaporated on a rotary evaporator. Finally, *Radix Scutellariae* extract, *Fructus Gardeniae* extract and *Pulvis Fellis Suis* were mixed and kept at the temperature of −20°C before use.

Liquid chromatograph mass spectrometer/mass spectrometer (LC-MS-MS) was used in a previous study to analyze the composition of the QZZD. It was implemented on an UHPLC system (Vanquish, Thermo Fisher Scientific) with a Waters UPLC BEH C18 column (1.7 μm 2.1 *100 mm). The flow rate was set at 0.4 ml/min and the volume of QZZD sample was set at 5 μl. The mobile phase consisted of 0.1% formic acid in water (A) and 0.1% formic acid in acetonitrile (B). The multi-step linear elution gradient program was as follows: 0–3.5 min, 95–85% A; 3.5–6 min, 85–70% A; 6–6.5 min, 70–70% A; 6.5–12 min, 70–30% A; 12–12.5 min, 30–30% A; 12.5–18 min, 30- 0% A; 18–5 min, 0–0% A; 25–26 min, 0–95% A; 26–30 min, 95–95% A. An Orbitrap Exploris 120 mass spectrometer coupled with an Xcalibur was employed to obtain the MS and MS/MS data based on the information dependent acquisition mode. And initially identified baicalin, geniposide, cholic acid, hyodeoxycholic acid, and chenodeoxycholic acid (Liu et al., 2022). The results of Ultra High Performance Liquid Chromatography Q Extractive Mass Spectrometry (UHPLC-QE-MS) for QZZD are shown in Supplementary Tables 2, 3.

#### Animals and drug administration

We chose two kinds of mice in this experiment where C57BL/6 mice were in the control group (*n* = 12), while a total of 36 APP/PS1 double transgenic mice were randomly divided into model group (*n* = 12, 0.9% saline solution, i.g.), QZZD group (*n* = 12, QZZD 45 mg/kg/d, i.g.), and DNZ (Shanghai, China) group (*n* = 12, donepezil 1.3 mg/kg/d, i.g.). It should be noted that APP/PS1 double transgenic mice present with abnormal expression of Aβ that will lead to extracellular deposition and plaque formation (Poisnel et al., 2012). The model was used before in AD drug test, and showed similar neuropathological and behavioral characteristics with AD (Pagnier et al., 2018). In this experiment, the mice were 12–13-week males weighing 28–30 g, and they were obtained from Huafukang Biotechnology [license No.: SCXK (Jing) 2019-0008] (Beijing, China). We kept the mice in an environment with temperature of 23 ± 2°C, relative humidity of 65% ± 5%, and 12/12 h light/dark cycle, and provided them with enough food and water.

The design and protocols of animal experiment were approved by the Ethics Review Committee for Animal Experimentation of Beijing University of Chinese Medicine (BUCM-4-2018122101-1018). The animals were euthanized through inhalation of excessive isoflurane after the water maze experiment. Their brains were immediately removed after sacrifice so that follow-up experiments could be carried out.

#### Weight analysis

After one week of adaptive feeding, the weight was recorded at the same time per week for subsequent statistical analysis.

#### Water maze experiment

Water maze experiment is a classic behavioral experiment aiming to detect spatial learning and memory ability and define changes of cognitive functions before and after administration in mice with dementia. The Morris Water Maze (MWM) test includes the place navigation test and the spatial probe test (Vorhees and Williams, 2006). After 8 weeks of continuous administration, mice in each group received behavior assessment in water maze at the experimental center of Beijing University of Chinese Medicine. The water maze system includes a circular pool with a diameter of 120 cm and a height of 50 cm, a hidden platform 10 cm in diameter and a video positioning instrument. The depth of water in the pool is 40 cm and the pool is divided into four quadrants. The hidden platform is submerged 1 cm below the water level in the target quadrant. Animal milk powder is added until the water surface turns milky white, while the periphery of the pool is covered by a black curtain. The water temperature is kept at 22 ± 2°C. The assessment lasted for 5 days, where place navigation test was conducted in the first 4 days, and spatial probe test was conducted on the last day.

(1) The place navigation test: the mice were placed into the pool from 4 quadrants successively with their heads toward the wall, and the time they spent reaching the hidden platform was recorded. If they failed to finish in 60 s, we guided them to the platform and allowed them to rest for 10 s. The test lasted for four continuous days.
(2) The spatial probe test: the hidden platform was removed on the 5th day, and the mice were put into the pool from the opposite of the target quadrant. The system automatically recorded the time when the mice first reached the platform within 60 s, the number of times they had crossed the original platform, the time they had stayed in the target quadrant, and the total moving distance within the specified time. EthoVision XT software (Noldus, China) was then used for analysis.

#### Hematoxylin and eosin staining analysis

Hematoxylin and eosin (HE) staining was used to observe neuronal morphology in the CA1 region of the mouse hippocampus. Mice were sacrificed and their brain tissues were immediately fixed in 4% paraformaldehyde solution. Afterward, the brain tissues were embedded in paraffin and cut into 5 μm slices. Then the slices were put on a slide and dried at 38°C for 24 h. The dried slices were dewaxed and rehydrated before being stained with hematoxylin for 5.5 min. Then they were rinsed in running tap water for 10 s, and finally stained with eosin for 8 s before dehydration and mounting.

#### Immunofluorescence analysis

After the end of administration, the freshly dissected brain tissue (7 μm) was fixed in formalin and embedded in paraffin for preparation of sections. The sections were then deparaffinized and rehydrated before being repaired in citrate buffer (pH 9.0) at the temperature of 80°C for 50 min. Then 3% hydrogen peroxide was used to quench endogenous peroxidase before being blocked with 10% goat serum in an incubator (37°C) for 30 min. Thereafter, the sections were incubated with anti-NeuN antibody (1:100, Abcam, MA, USA) at the temperature of 4°C for 48 h. The secondary incubation was conducted with goat anti-mouse DyLight 488 antibody (1:200, Abcam, MA, USA) at room temperature (37°C) for 2 h. The next step was DAPI staining, which is followed by quenching of fluorescence and mounting. After that, morphology of sections was observed under a laser scanning confocal fluorescence microscope and photographed at the indicated sites. Quantified analysis was performed to positive cells present in the sections at lens of 20 × and 40 ×, respectively, and the number of NeuN-positive cells was calculated in a random microscope field of each slice by CellF software.

#### Western blotting analysis

After the end of administration, the hippocampal tissues of the mice were collected before lysis in RIPA buffer on ice. The supernatant was acquired after centrifugation. Then protein concentration of the sample was determined using bicinchoninic acid (BCA) assay, which was followed by protein separation by sodium dodecyl sulfate-polyacrylamide denaturing gel electrophoresis (SDS-PAGE). The next step was transferring the separated proteins from the gel onto polyvinylidene fluoride (PVDF) membrane with wet electroblotting. The membrane was blocked with 5% bovine serum albumin (BSA) in a mixture of Tris-buffered saline and Tween-20 (TBST) for 1 h. Then it was incubated with β-actin (1:1000) and primary antibodies against TNFR1 (1:1000, Abcam, MA, USA), NF-κB p65 (1:2000, Abcam, MA, USA), ERK1/2 (1:1000, Abcam, MA, USA), p-ERK1/2 (1:1000, Abcam, MA, USA), IL-6 (1:1000, Abcam, MA, USA), Aβ (1:1000, Abcam, MA, USA) overnight at 4°C. Then the blots were incubated with the secondary antibody at room temperature for 1 h. When the immunodetection was finished, ECL assay kit (Nanjing, China) was used to visualize the protein bands which were then analyzed by Gel Image software version 4.00 (Tanon, China).

#### Immunohistochemical analysis

After a series of routine manipulations including deparaffinization, rehydration, repair and blocking as in 2.2.7, the sections were incubated with antibodies against Aβ (1:150, Abcam, MA, USA), TNFR1 (1:1000, Abcam, MA, USA), NF-κB p65 (1:200, Abcam, MA, USA), and IL-6 (1:300, Abcam, MA, USA) at 4°C overnight. Then biotin-labeled secondary antibodies were used for the second incubation at room temperature (37°C) for 20 min. The next step was 3,3′-Diaminobenzidine (DAB) staining, which was followed by hematoxylin staining of the nuclei. Then the sections were dehydrated and stabilized with mounting medium. After that, the staining area and the average optical density were calculated, and Image-Pro Plus 6.0 software (Media Cybernetics, MD, USA) was used for analysis.

#### Statistical analysis

SPSS (version 20.0, IBM, Armonk, NY) was used for statistical analysis. Data were expressed as mean ± standard deviation (SD). Experimental data of each group were analyzed by normality test and homogeneity of variance test. One-Way Analysis of Variance (ANOVA) (Bewick et al., 2004) was used for statistical tests among multiple groups, and Fisher’s Least Significant Difference (Meier, 2006) was used for comparison between groups. Those with *P* value less than 0.05 were considered to be statistically significant.

## Results

### Network pharmacology analysis of Qin-Zhi-Zhu-Dan Formula

#### Putative targets of Qin-Zhi-Zhu-Dan Formula against Alzheimer’s disease

According to the four principles (*MW* < *500*, *AlogP* < *5*, *Hdon* < *5*, and *Hacc* < *10*), a total of 15 active compounds of QZZD were identified through TCMSP database in combination with text mining in Google Scholar, CNKI, and PubMed. See Supplementary Table 4 for detailed information. Then a total of 521 non-repeated QZZD targets (Supplementary Table 5) were obtained from TCMSP and normalized with reference to the UniProt database.

**FIGURE 2:**
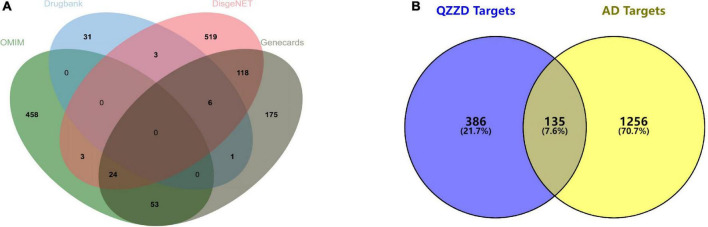
Venn diagram. **(A)** Venn diagram of AD-related targets from the DisGeNET database, OMIM database, Drugbank database and GeneCards database. **(B)** Venn diagram of 135 common targets between QZZD and AD.

A total of 1,391 AD-related targets were collected from the DisGeNET database, OMIM database, Drugbank database and GeneCards database (Figure 2A and Supplementary Table 6). There were 135 common targets of neuroinflammation identified in the Venn diagram (Figure 2B), which were putative targets of QZZD against AD.

#### The compound-target network

**FIGURE 3:**
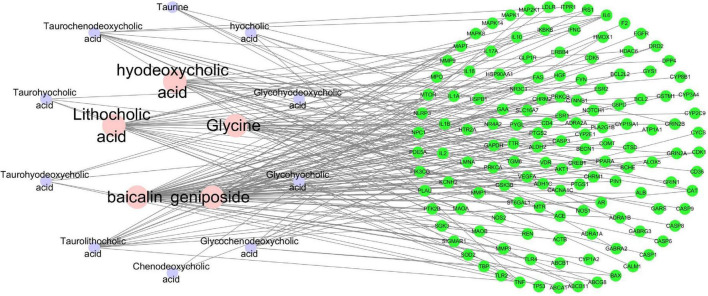
The Compound-Target Network of the 14 compounds and the 135 common targets. The nodes represent active compounds and target genes, and the edges represent the interaction between them. The degree value of the node represents the number of interactions.

Cytoscape is a popular application (Shannon et al., 2003) that can analyze and visualize graph-oriented genomic networks and has been widely used in network pharmacology studies. In this study, Cytoscape 3.7.1 software was used for visualization of compound-target network of the 14 compounds (glycohyodeoxycholic acid was excluded for lack of potential therapeutic targets) and the 135 common targets (Figure 3). The network contains 149 nodes and 200 edges, with an average node degree of 2.685. With CytoHubba, compounds including baicalin, geniposide, lithocholic acid, glycine, and hyodeoxycholic acid with the highest degree value were considered to be important active compounds.

#### Protein-protein interaction network

**FIGURE 4:**
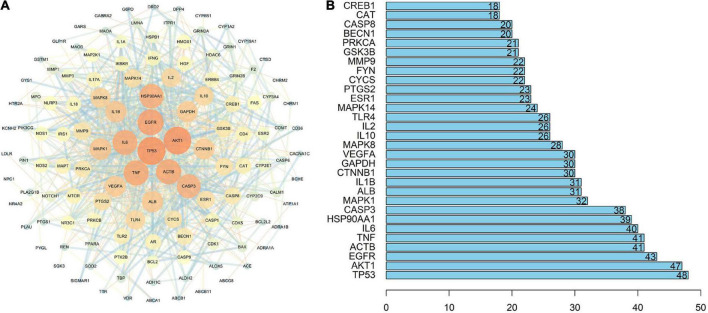
Protein-Protein Interaction (PPI) network. **(A)** PPI network of the 135 common targets between QZZD and AD. Nodes represent target genes, and edges represent their interactions. The size and color of nodes are related to the value of degree. With increase of the degree, the size of nodes grows and the color gradually changes from blue to orange. The thickness and color of the edges are related to the combined score value. With increase of the combined score, the thickness of edges grows and the color gradually changes from orange to blue. **(B)** Core targets of PPI network. The numbers inside the bars represent the degree value of each target.

The STRING database (Szklarczyk et al., 2019) was used to determine protein-protein interactions of common target genes between QZZD and AD. Those with an interaction score greater than 0.7 were entered into Cytoscape 3.7.1 for PPI network analysis and visualization (Figure 4A). The network consisted of 135 nodes and 822 edges. The correlation topological analysis yields an average node degree value of 12.2 and an average local clustering coefficient of 0.467.

Core targets, that are key nodes in the network, can be used to assess the nature of the entire network, and are often identified by the degree value. With the help of R software (version 4.0.3), the top 30 targets genes of the highest degree value were obtained, including tumor protein p53 (TP53), serine/threonine-protein kinase (AKT1), epidermal growth factor receptor (EGFR), beta cytoskeletal actin (ACTB), tumor necrosis factor (TNF), interleukin-6 (IL-6) and so on, which served as core targets in the network (Figure 4B).

#### Gene Ontology analysis of putative targets

**FIGURE 5:**
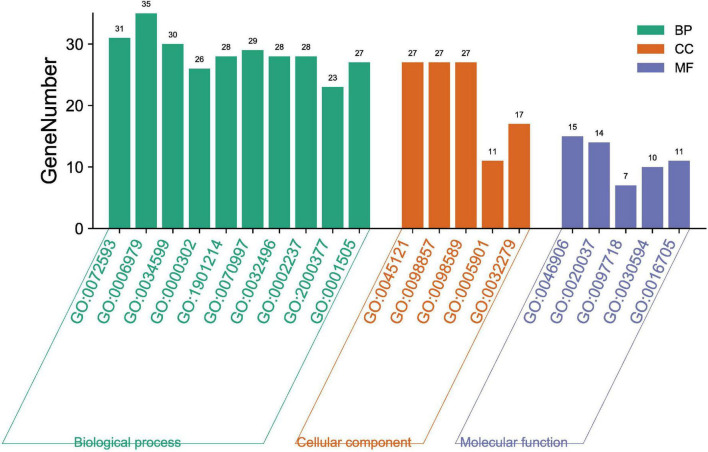
GO enrichment analysis of putative targets of QZZD against AD.

The biological functions of the 135 targets were clarified through GO enrichment analysis. A total of 182 results were obtained, which were then classified and annotated using the Gene Ontology annotation (GOA) database in terms of molecular function (MF), cell component (CC) and biological process (BP). There were 5 related to MF, 5 to CC and 10 to BP according to *p* value (Figure 5). To be more specific, QZZD mainly participated in the following 10 BPs, including reactive oxygen species (ROS) metabolic process (GO:0072593), response to oxidative stress (GO:0006979), cellular response to oxidative stress (GO:0034599), response to reactive oxygen species (GO:0000302), regulation of neuron death (GO:1901214), neuron death (GO:0070997), response to lipopolysaccharide (GO:0032496), response to molecule of bacterial origin (GO:0002237), regulation of reactive oxygen species metabolic process (GO:2000377), and regulation of neurotransmitter levels (GO:0001505). Besides, QZZD affected AD by regulating two principal MFs, namely, tetrapyrrole binding (GO:0046906) and heme binding (GO:0020037). When it comes to CC, target proteins of QZZD might exist in membrane raft (GO: 0045121), membrane microdomain (GO: 0098857), and membrane region (GO: 0098589).

#### Target-pathway analysis of Qin-Zhi-Zhu-Dan Formula

**FIGURE 6:**
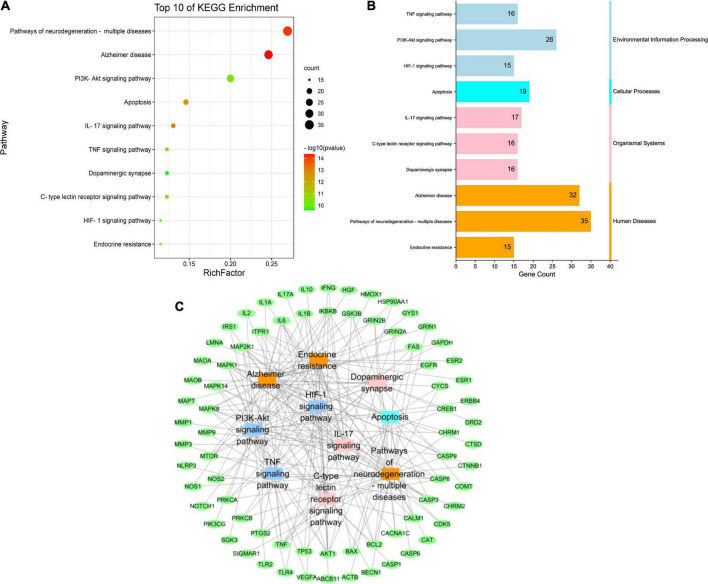
KEGG pathway enrichment analysis of putative targets of QZZD. **(A)** Bubble diagram of the top 10 pathways. **(B)** KEGG pathway classification. **(C)** Target-pathway network. Green nodes refer to target proteins. Dark blue nodes represent pathways of environmental information processing. Light blue node represents pathway of cellular processes. Pink nodes represent pathways of organismal systems. Orange nodes represent pathways of human diseases.

Through KEGG pathway enrichment analysis, we attempted to identify the key pathways associated with the 135 putative targets, and a total of 197 pathways were obtained. After excluding unrelated pathways, such as “human cytomegalovirus infection,” “tuberculosis” and “hepatitis B,” the top 10 signaling pathways with the lowest *p* value were selected (Figure 6A), among which, the PI3K-Akt, TNF, IL-17 and HIF-1 signaling pathways were correlated with inflammation and immunity, while apoptosis, Alzheimer’s disease, pathways of neurodegeneration-multiple diseases, and dopaminergic synapse were closely related to the pathogenesis of AD. According to KEGG pathway classification^13^, they were divided into four categories, namely, environmental information processing, cellular processes, organismal systems and human diseases (Figure 6B). Through construction of target-pathway network (Figure 6C), it was found that some pathways shared the same targets. For example, the PI3K-Akt signaling pathway and the TNF signaling pathway shared the targets of RAC-alpha serine/threonine-protein kinase (AKT1), cyclic AMP-responsive element-binding protein 1 (CREB1), inhibitor of nuclear factor kappa-B kinase subunit beta (IκBKB), IL6, dual specificity mitogen-activated protein kinase kinase 1 (MAP2K1), and mitogen-activated protein kinase 1 (MAPK1). The discovery that some genes could participate in multiple pathways simultaneously indicated that these pathways were highly correlated and complicated.

### Integrated pathway analysis

**FIGURE 7:**
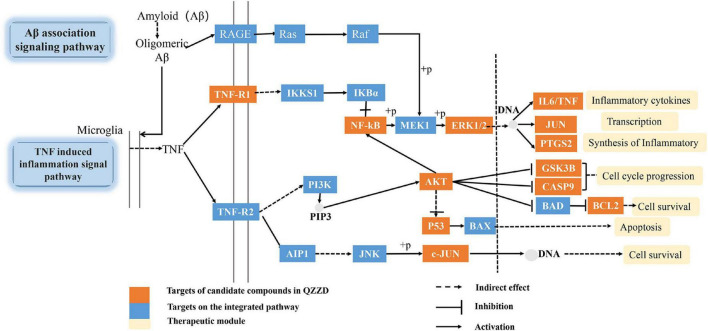
AD-integrated pathway (+p represents phosphorylation).

On the basis of the above analysis of pathway correlation and core targets, we mainly focused on the anti-inflammatory mechanism of QZZD in treating AD and assembled it into a synthetic pathway (Figure 7) with TNF signaling pathway as the main pathway, including Aβ-associated signaling pathway, NF-kappaB signaling pathway and PI3K-Akt signaling pathway. Abnormal Aβ aggregation plays a crucial role in the pathogenesis of AD, and it can trigger other pathological processes, such as mitochondrial dysfunction and inflammation, which ultimately leads to neuronal death (Montgomery and Bowers, 2012). The proinflammatory cytokine tumor necrosis factor-α (TNF-α) is a pivotal molecule in inflammation (Simpson and Ferguson, 2017). Studies using genetic and pharmacological manipulations *in vivo* confirmed that Aβ and tau pathologies could be worsened by TNF-α signaling (Decourt et al., 2017). Extracellular regulated protein (ERK1/2), p38 MAPK, and c-Jun N-terminal kinase (JNK) are three major MAPKs that regulate a wide variety of cellular processes and play a crucial role in inhibiting the expression of proinflammatory cytokines such as TNF-α and interleukin-1β (IL-1β) (Jayaraj et al., 2019). In conclusion, QZZD might prevent and treat AD by regulating neuroinflammation.

### Experimental validation

#### Effects of Qin-Zhi-Zhu-Dan Formula on weight change

**FIGURE 8:**
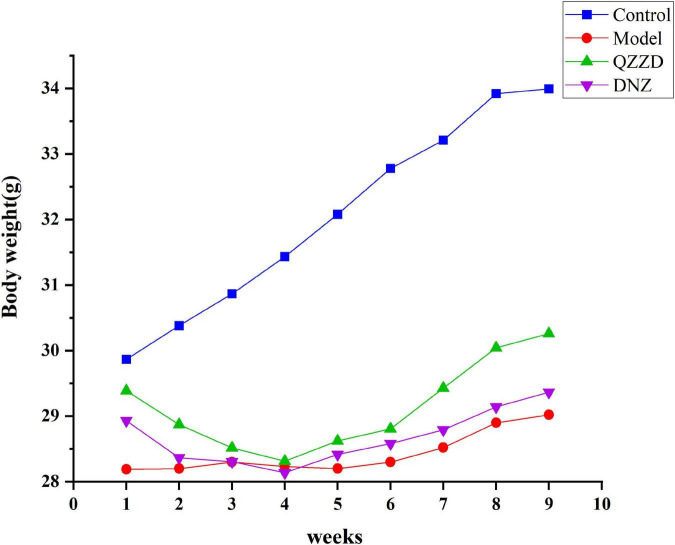
Weight change of mice in different groups. Values are expressed as mean ± SD, *n* = 12 in each group.

Weight change of mice in the control group differed from those in the other groups because the mice were from another strain, while the rest were APP/PS1 double transgenic mice. In the model group, there was a slight increase in the second and third week, which was then followed by a slight decrease in the fourth week; the weight fluctuated slightly in the first 3 weeks, and then increased gradually, with a growth rate lower than those of QZZD group and DNZ group. QZZD group showed the same weight change as that of DNZ group in the first 4 weeks where the weight fluctuated greatly in a downward manner in the second, third and fourth week. There was an increase in weight in both groups from the fifth week, with a higher growth rate in QZZD group (Figure 8).

#### Effects of Qin-Zhi-Zhu-Dan Formula on spatial learning and memory

**FIGURE 9:**
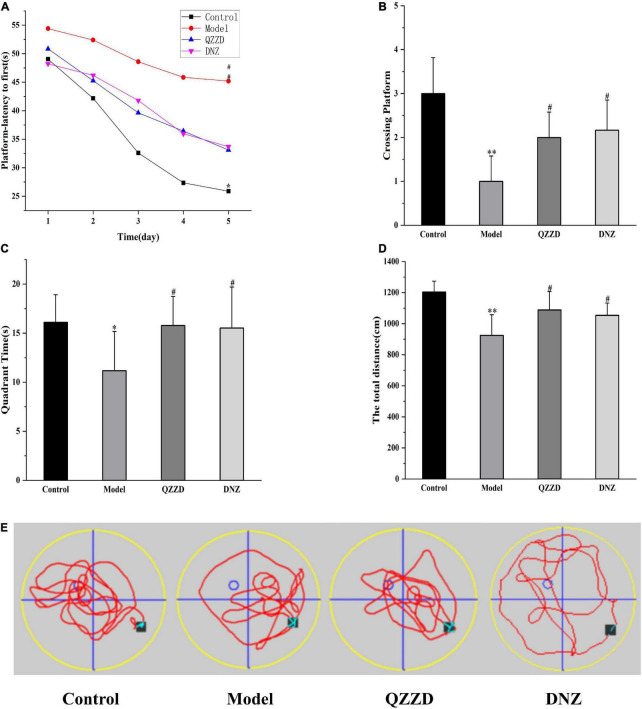
Effects of QZZD on spatial learning and memory after 8 weeks of treatment. **(A)** Escape latency to hidden platform. **(B)** Number of crossing the original platform. **(C)** Time spent in the target quadrant. **(D)** The total moving distance. **(E)** Representative swimming path of mice. Values are expressed as mean ± SD, *n* = 12 in each group. *p < 0.05, ^**^p < 0.01 compared with the control group; #*p* < 0.05 compared with the model group.

The APP/PS1 double transgenic mouse model is commonly used in studies on cerebral and neurological disorders, especially in AD (Chen C. L. et al., 2022), amyloid plaque formation, and aging (Ordóñez-Gutiérrez et al., 2013). In the place navigation test, escape latency and trend line can effectively reflect changes of learning and memory function of mice in each group. Compared with the control group, the escape latency of mice in the model group was significantly increased from the second day of the MWM, especially on Day 4 and Day 5 (Figure 9A). It was also found that the spatial learning performance of mice treated with QZZD and DNZ was significantly improved. During the probe test, mice in the model group crossed the original platform less frequently, spent less time in the target quadrant, and the swimming path is more complicated than that in the control group (Figures 9B,C). It could also be observed that the paths of mice in the control group and QZZD group were similar (The mice did not lose the way and made similar attempts, Figure 9E), which was not seen in DNZ group. This indicated that QZZD was more effective than DNZ. The total moving distance of mice in the model group was significantly lower than that in the control group, QZZD group and DNZ group (Figure 9D). In conclusion, QZZD could significantly improve spatial learning and memory in APP/PS1 double transgenic mouse with dementia.

#### Effects of Qin-Zhi-Zhu-Dan Formula on neuronal death

**FIGURE 10:**
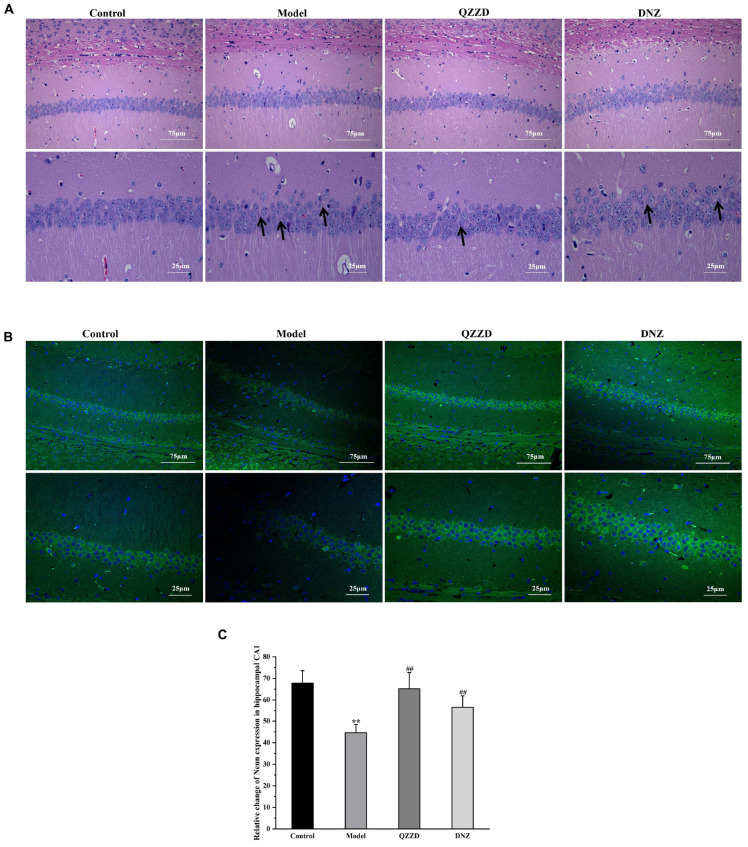
Effects of QZZD on neuronal death. **(A)** CA1 region of the hippocampus after HE staining [Scale bar: 75 and 25 μm (insert), *n* = 6]. **(B)** CA1 region of the hippocampus after NeuN staining [Scale bar: 75 and 25 μm (insert)]. **(C)** Number of NeuN-positive cells in CA1 region of the hippocampus. Values were expressed as means ± SD, *n* = 6 in each group. **p < 0.01, compared with the control group; ##*p* < 0.01, compared with the model group.

Neurons in the hippocampus of the mice were examined with HE and NeuN staining. According to the results of HE staining, the hippocampal neurons in the control group showed normal morphologies and clear boundaries. In contrast, neurons in the model group manifested with abnormal morphologies. For example, the area indicated by the arrows showed shrunken nuclei of pyramidal cells and even missing/lost cells. It was also found that such neuronal damage was reduced in QZZD and DNZ groups (Figure 10A). Besides, QZZD was effective in restoring the decreased number of neurons in CA1 region of the hippocampus in mice with dementia (Figures 10B,C). These findings suggested that QZZD could attenuate neuronal death in the CA1 region of the hippocampus in mice with dementia.

#### Effects of Qin-Zhi-Zhu-Dan Formula on neuroinflammation

**FIGURE 11:**
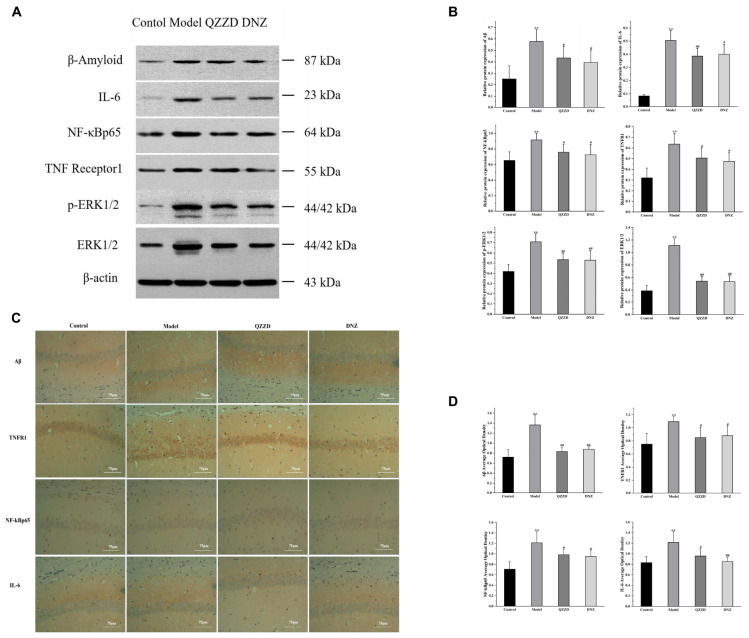
Effects of QZZD on expressions of Aβ, IL-6, NF-κBp65, TNFR1, p-ERK1/2 and ERK1/2 in hippocampal tissues of APP/PS1 double transgenic mice with dementia. **(A)** Western blotting analysis showing the protein expression levels of Aβ, IL-6, NF-κBp65, TNFR1, p-ERK1/2 and ERK1/2 in the hippocampus. **(B)** Quantitative analysis of Aβ, IL-6, NF-κBp65, TNFR1, p-ERK1/2, and ERK1/2 protein bands. Values are expressed as the mean ± SD, *n* = 6 in each group. **p < 0.01, compared with the control group; #*p* < 0.05, ##*p* < 0.01, compared with the model group. **(C)** Immunohistochemistry analysis showing the protein expression levels of Aβ, TNFR1, NF-κBp65, and IL-6 in the hippocampus. **(D)** The average optical density of Aβ, TNFR1, NF-κBp65 and IL-6 in the hippocampus. Values are expressed as the mean ± SD, *n* = 6 for each group. **p < 0.01, compared with the control group; #*p* < 0.05, ##*p* < 0.01, compared with the model group.

The effects of QZZD on mice with dementia were determined by the levels of protein expression of core targets in the TNF inflammatory signaling pathway in hippocampal tissue. Six core targets, including Aβ, IL-6, NF-κBp65, TNFR1, ERK1/2, and p-ERK1/2, were further selected for Western blotting analysis. According to the Western blotting results in Figures 11A,B, the expressions of Aβ, IL-6, NF-κBp65, TNFR1, ERK1/2, and p-ERK1/2 in the model group were significantly increased compared with those in the control group (*p* < 0.01). After 8 weeks of intervention, the protein levels in QZZD and DNZ groups were significantly decreased compared with those in the model group. For QZZD group, the expressions of IL-6, ERK1/2 and p-ERK1/2 were decreased significantly (*p* < 0.01), and the expressions of Aβ, TNFR1 and NF-κBp65 were also inhibited (*p* < 0.05) in QZZD compared with those in the model group. Besides, the levels of proteins in QZZD group were close to those in the control group, which indicated that QZZD tended to restore the levels of these proteins. Moreover, we selected several major protein indicators, including Aβ, TNFR1, NF-κBp65, and IL-6, for immunohistochemical examination (Figures 11C,D), and the results were consistent with those of the Western blotting. In conclusion, the results of Western blotting and immunohistochemical assays further confirmed the discoveries in the above network pharmacology analysis.

## Discussion

Great efforts have been put into studies on pathogenesis and treatment of AD. In this paper, we applied the network pharmacology approach to explore the molecular mechanism of QZZD in the treatment of AD, and combined experiments to further validate the results. It was found through network pharmacology analysis that TNF signaling pathway played an important role with targets of TNF, TNFR1, c-JUN, MAPK3, IL6, PTGS2, GSK3B, CASP9, BCL2, TP53, and AKT. We found through experiment *in vivo* that QZZD could protect the neurons, reduce damage to neural function, and enhance spatial learning and memory function in APP/PS1 double transgenic mice with dementia. The effects of QZZD might be related to regulation of the TNFR1-ERK1/2-NF-κBp65 inflammation pathway.

There is a lot of evidence confirming the importance of chronic neuroinflammation in the pathogenesis of AD (Liang et al., 2022). Effective control of inflammation can reduce the occurrence and delay the progression. Through network pharmacology and LC-MS-MS analyses, we identified the important active compounds of QZZD, such as baicalin, geniposide, lithocholic acid, glycine, hyodeoxycholic acid and cholic acid. Among them, baicalin (Li et al., 2020), geniposide (Huang et al., 2018), and glycine (Ullah et al., 2020) could significantly influence neuroinflammation, which suggested that QZZD might reduce neuroinflammation.

To further investigate the underlying mechanism of QZZD against AD, PPI network analysis was carried out to predict the potential targets of QZZD on AD. In this study, the 135 common targets between QZZD and AD were used to construct the PPI network, among which there were six core targets (TP53, AKT1, EGFR, ACTB, TNF, IL-6) according to the degree value. TP53 is an important tumor suppressor gene which plays a key role in regulating apoptosis, senescence, and DNA damage repair in response to cellular stress (Granowicz and Jonas, 2022). Recently, it was found that TP53-induced glycolysis and apoptotic regulators (TIGAR) might protect the neurons by inhibiting oxidative stress and neuroinflammation (Li Q. Q. et al., 2021; Huang et al., 2022). AKT1, member of the AKT family, regulates essential functions such as cell survival, proliferation, and angiogenesis (Alwhaibi et al., 2019). Inhibiting phosphorylation of AKT1 can induce BV-2 microglia activation which leads to neuroinflammation (Xiao et al., 2022). EGFR is one of the receptor proteins of the growth factor family, and participates in cell proliferation and signal transduction (Shi et al., 2022). Inhibition of EGFR/MAPK signaling pathway could suppress microglia activation and reduce secondary damage related to neuroinflammation (Qu et al., 2012). There was stable expression of ACTB gene in cell lines and multiple human tissues. However, in the case of certain diseases, ACTB expression might experience drastic changes in the whole blood, which leads to changes in cell composition of the peripheral immune system (Stamova et al., 2009; Sieber et al., 2010). Recent study showed that preclinical altered blood-based ACTB methylation had a significant correlation with stroke, which suggested that it might be a potential regulatory indicator for early detection and even prevention of stroke (Liu et al., 2021). However, whether ACTB has diagnostic significance in AD remains unclear. TNF superfamily are the key mediators of neuroinflammation. It was found that TNF-mediated neuroinflammation is linked to neuronal necroptosis in AD (Jayaraman et al., 2021). IL-6 is a combination of proinflammatory cytokines and anti-inflammatory cytokines and has an important role in the nervous system. It can intensify expression of downstream proinflammatory factors, promote the oxidative stress response for brain tissue damage, neuronal cell apoptosis or atrophy, promote Aβ precipitation, and affect the integrity of neuronal axons or dendritic membrane. The increase in IL-1β and IL-6 levels can promote inflammatory response and local oxidative stress disorder, which leads to the deposition of beta-twisted proteins in neuronal interstitial components and the apoptosis of neurons. Similarly, it can also promote the phagocytosis of NK cells or glial cells and aggravates the decline of neuronal functions in AD patients.

Based on the 135 common targets, we also conducted GO enrichment analysis and KEGG enrichment analysis. GO enrichment analysis showed that oxidative stress and neuron death were important biological processes in treatment of AD. In terms of oxidative stress, although it is pathologically different from neuroinflammation in neurodegenerative diseases, such as AD, they are also correlated. Oxidative stress can modify the inflammatory response, and it has been known that some ROS and reactive nitrogen species (RNS) could promote intracellular signaling cascades, which leads to increased expression of pro-inflammatory genes (Teleanu et al., 2022). The other biological process–neuronal loss in specific areas of the brain underlies the pathology of AD. It is well recognized that Aβ is the toxic species in AD and can induce neuron death (Akhter et al., 2014). Aβ accumulation can further aggravate neuroinflammation and lead to neuronal injury, which indicates that the reduction of Aβ accumulation is vital in reducing neuroinflammation, neuronal injury and death (Yan et al., 2022). KEGG enrichment analysis showed that the pathways related to inflammation and immunity, such as PI3K-Akt, TNF, IL-17 signaling pathways were correlated with the specific mechanism of QZZD in the treatment of AD. Meanwhile, the pathway of Alzheimer’s disease was also enriched in this KEGG analysis, which further indicated that QZZD could be used to treat AD.

After the network pharmacology analysis, we evaluated the effects of QZZD by experiment *in vivo* from the following three aspects. Firstly, the effects of QZZD on the digestive system were explored through weight change of the mice during administration; secondly, the spatial learning and memory ability of the mice were clarified through the water maze experiment; thirdly, the pathological and morphological changes in the hippocampus were examined by HE staining and immunofluorescence analysis. It was found that QZZD could not only alleviate impairments in spatial learning and memory but also attenuate neuronal death. Furthermore, we also observed that the development of AD might affect the digestive system of mice as the model group presented with decreased appetite and slow weight growth. The weight growth rate of mice in the QZZD group was higher than that in the DNZ group, which also confirmed that donepezil hydrochloride could affect the digestive system.

According to the network pharmacology analysis, we mainly focused on the anti-inflammatory mechanism of QZZD in treating AD and assembled it into a synthetic pathway (Figure 7), with the TNF signaling pathway as the main pathway, including the Aβ-associated signaling pathway, NF-kappaB signaling pathway and PI3K-Akt signaling pathway. Then we used Western blotting and immunohistochemistry analysis for the core targets, including TNFR1, NF-kBp65, ERK1/2, p-ERK1/2, IL-6 and Aβ, to further validate the mechanism. Western blotting analysis showed that, compared with the control group, the protein levels of Aβ, TNFR1, ERK1/2, p-ERK1/2, NF-κBp65 and the inflammatory cytokine IL-6 was significantly higher in the model group. Compared with the model group, the intervention treatment of QZZD and donepezil hydrochloride could significantly decrease the expression levels of Aβ, TNFR1, ERK1/2, p-ERK1/2, NF-κBp65 and the inflammatory cytokine IL-6, which confirmed the therapeutic effects of both interventions. The results of immunohistochemical analysis showed that compared with the model group, the staining region and optical density of Aβ, TNFR1, NF-kBp65 and IL-6 were significantly different in CA1 region of the hippocampus in the control group.

In this study, the central nervous system (CNS) immune cascade in AD model mice was induced by inflammatory factors and their reactions, as a result of which immunogens were formed, and then activated NF-κB in the brain, and further promoted secretion and release of TNF-α, IL-6 and other inflammatory factors. In the process ROS was also released and mediated the inflammatory response. Thus a cycle was formed. The chronic inflammatory response produced direct or indirect toxic effects on neuronal cells, eventually leading to the development of AD. Network pharmacology analysis and experiments confirmed that QZZD regulates the TNFR1-ERK1/2-NF-κBp65 inflammation pathway in the treatment of AD. It is of great significance to study regulation of inflammatory mechanisms, which can help to understand how to reduce pathological apoptosis of neurons through modulating neuroinflammation in the brain.

## Conclusion

In this paper, we comprehensively analyzed the mechanism of QZZD against AD combining network pharmacology analysis and experimental validation. In the first part, we identified 15 important compounds of QZZD, and 135 putative targets of QZZD against AD. GO analysis and KEGG enrichment analysis revealed the anti-inflammatory mechanism of QZZD, and a synthetic pathway–TNFR1-ERK1/2-NF-κBp65 signaling pathway was obtained. When it comes to the experiment, it was found that QZZD could not only alleviate impairments in spatial learning and memory, but also improve neuroinflammation and attenuate neuronal death without influencing the digestive system in APP/PS1 double transgenic mice with dementia. These findings further confirmed that QZZD had therapeutic effects on AD through inhibiting inflammatory response in regulating the synthetic signaling pathway. The formula is characterized by multi-target and multi-pathway action and comprehensive regulation, which might provide reference for regulatory mechanism of treating dementia.

## Data availability statement

The datasets presented in this study can be found in online repositories. The names of the repository/repositories and accession number(s) can be found in the article/Supplementary material.

## Ethics statement

The animal study was reviewed and approved by the Ethics Review Committee for Animal Experimentation of Beijing University of Chinese Medicine (BUCM-4-2018122101-1018).

## Author contributions

QW, FC, and XW conceived the research theme. WX and BR participated in the research design and wrote the manuscript. ZZ and CM performed target prediction and analysis as well as related enrichment processes. SL and CC contributed to animal experiments. TX conducted the data analysis. XW revised the language and supervised the implementation. All authors have read and approved the final manuscript.

## Footnotes

1
https://old.tcmsp-e.com/tcmsp.php

2
https://scholar.google.com/

3
http://www.cnki.net/

4
https://www.ncbi.nlm.nih.gov/pubmed/

5
https://www.drugbank.ca/

6
https://www.ncbi.nlm.nih.gov/omim

7
https://www.disgenet.org/

8
http://www.genecards.org/

9
https://www.uniprot.org/

10
http://bioinfogp.cnb.csic.es/tools/venny/index.html

11
https://string-db.org/

12
http://www.r.project.org/

13
https://www.kegg.jp/kegg/pathway.html
