# Correction: Network pharmacology analysis reveals neuroprotective effects of the Qin-Zhi-Zhu-Dan Formula in Alzheimer's disease

**DOI:** 10.3389/fnins.2025.1752611

**Published:** 2025-12-04

**Authors:** Wenxiu Xu, Beida Ren, Zehan Zhang, Congai Chen, Tian Xu, Shuling Liu, Chongyang Ma, Xueqian Wang, Qingguo Wang, Fafeng Cheng

**Affiliations:** 1 School of Traditional Chinese Medicine, Beijing University of Chinese Medicine, Beijing, China; 2 Dongzhimen Hospital, Beijing University of Chinese Medicine, Beijing, China; 3 Institute for Brain Disorders, Beijing University of Chinese Medicine, Beijing, China; 4 School of Traditional Chinese Medicine, Capital Medical University, Beijing, China

**Keywords:** Alzheimer's disease, Qin-Zhi-Zhu-Dan Formula, TNF signaling pathway, network pharmacology, inflammatory response

There was a mistake in Figure 10B as published. The higher magnification images in the QZZD and DNZ columns were incorrectly swapped: the image labeled QZZD belonged to the DNZ group, and the image labeled DNZ belonged to the QZZD group. The corrected Figure 10 appears below.

**Figure 10:**
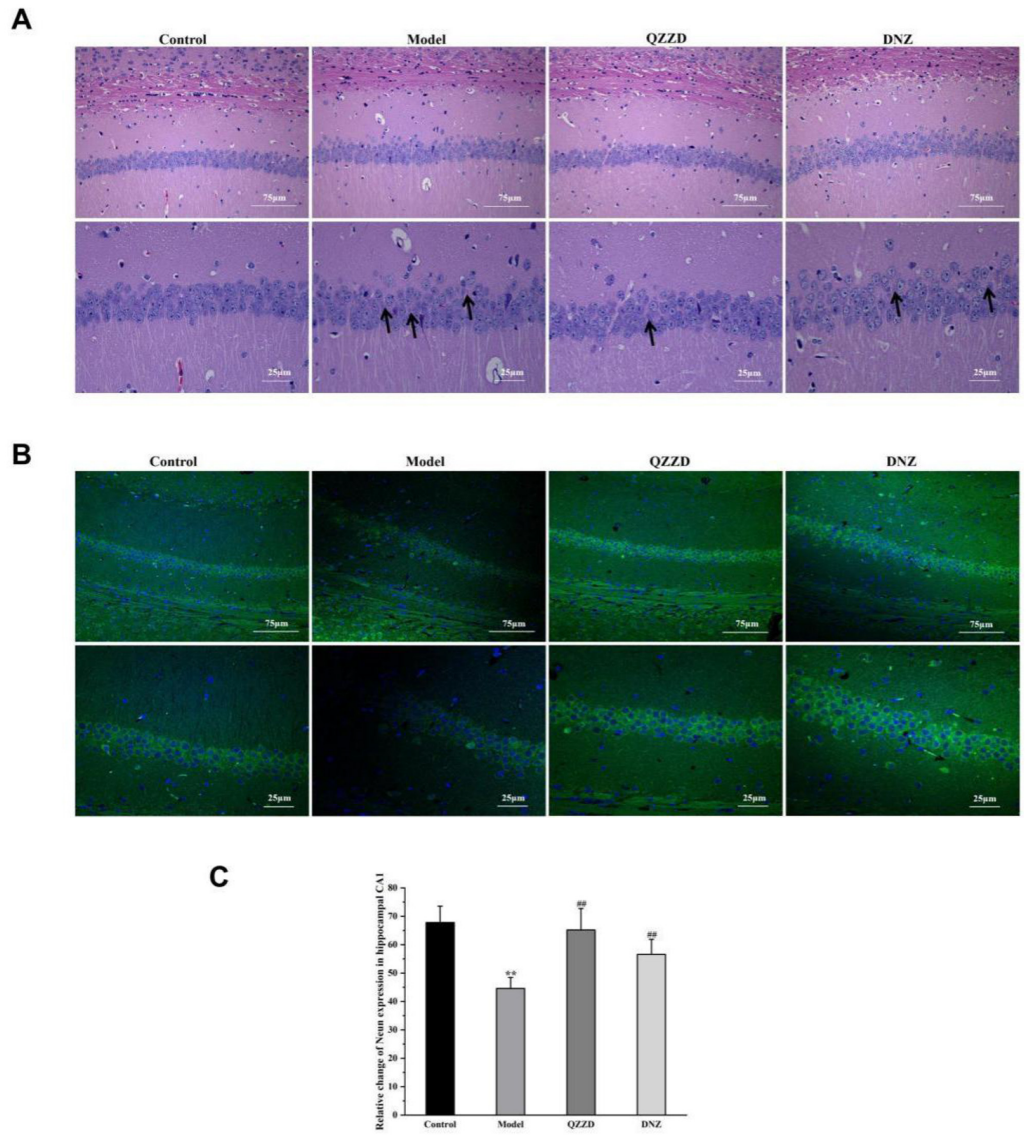
Effects of QZZD on neuronal death. **(A)** CA1 region of the hippocampus after HE staining [Scale bar: 75 and 25 μm (insert), *n* = 6]. **(B)** CA1 region of the hippocampus after NeuN staining [Scale bar: 75 and 25 μm (insert)]. **(C)** Number of NeuN-positive cells in CA1 region of the hippocampus. Values were expressed as means ± SD, *n* = 6 in each group. ^**^p < 0.01, compared with the control group; ^##^*p* < 0.01, compared with the model group.

There was a mistake in Figure 11C as published. An image designated for the QZZD group was incorrectly reused in both QZZD and DNZ panels. The corrected Figure 11 appears below.

**Figure 11:**
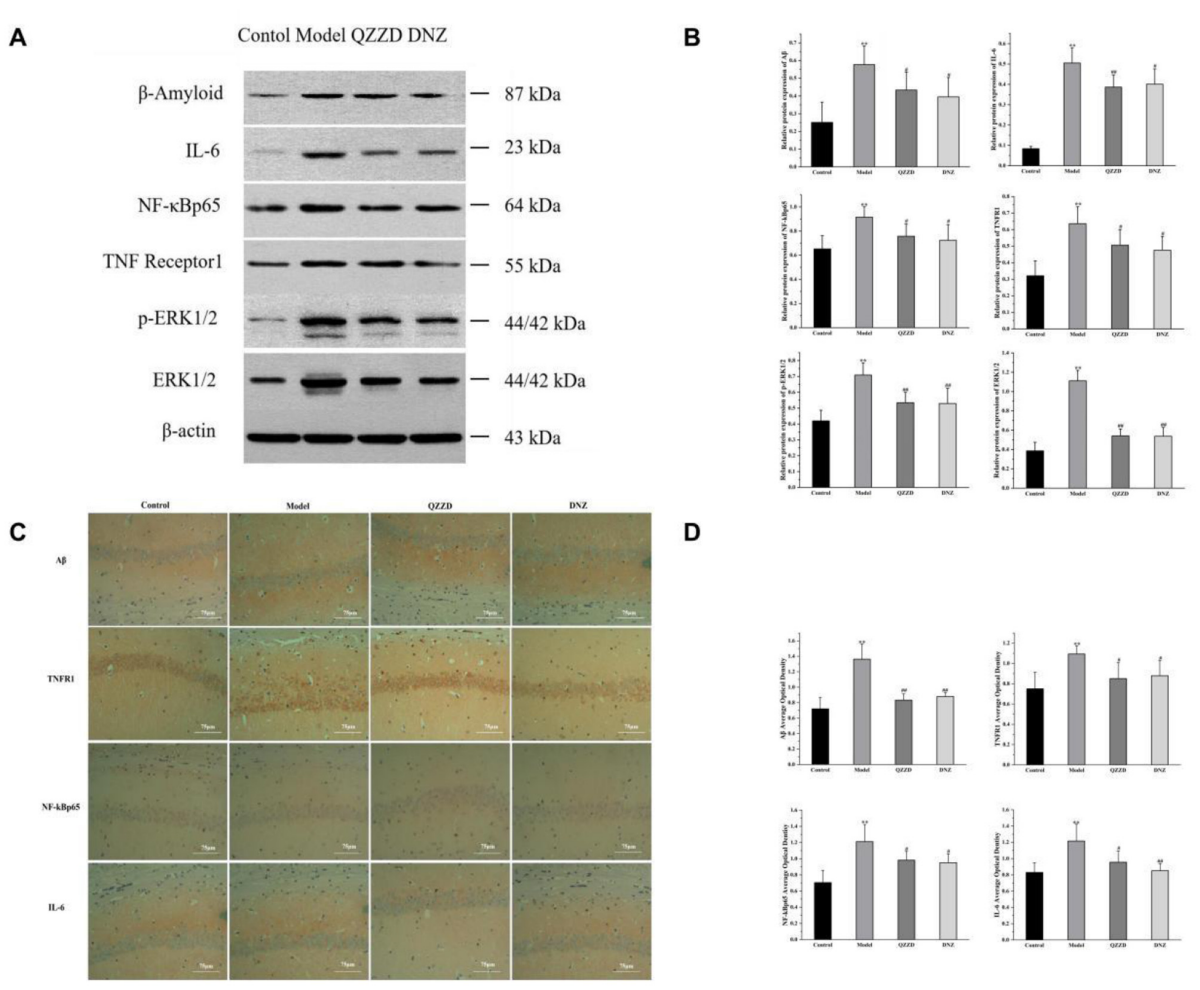
Effects of QZZD on expressions of Aβ, IL-6, NF-κBp65, TNFR1, p-ERK1/2 and ERK1/2 in hippocampal tissues of APP/PS1 double transgenic mice with dementia. **(A)** Western blotting analysis showing the protein expression levels of Aβ, IL-6, NF-κBp65, TNFR1, p-ERK1/2 and ERK1/2 in the hippocampus. **(B)** Quantitative analysis of Aβ, IL-6, NF-κBp65, TNFR1, p-ERK1/2, and ERK1/2 protein bands. Values are expressed as the mean ± SD, *n* = 6 in each group. ^**^p < 0.01, compared with the control group; ^#^*p* < 0.05, ^##^*p* < 0.01, compared with the model group. **(C)** Immunohistochemistry analysis showing the protein expression levels of Aβ, TNFR1, NF-κBp65, and IL-6 in the hippocampus. **(D)** The average optical density of Aβ, TNFR1, NF-κBp65 and IL-6 in the hippocampus. Values are expressed as the mean ± SD, *n* = 6 for each group. ^**^p < 0.01, compared with the control group; ^#^*p* < 0.05, ^##^*p* < 0.01, compared with the model group.

The original version of this article has been updated.

