# Supplementary material for: Network pharmacology analysis reveals neuroprotective effects of the Qin-Zhi-Zhu-Dan Formula in Alzheimer’s disease
Source: Front Neurosci. 2022 Oct 20;16:943400. doi: 10.3389/fnins.2022.943400 (PMC9632440; doi:10.3389/fnins.2022.943400)
Supplement: Supplementary file 3 [file Table_3.pdf]

Table S3.Tentative identification of the main peaks in QZZD in positive mode

Name	Formula	Class	t/min
(10E,12E)-9-hydroxyoctadeca -10,12-dienoic acid	C18H32O3	Aliphatic acyl	12.8
18 beta-Glycyrrhetintic Acid	C30H46O4	Terpenoids	13.74
3-Formylindole	C9H7NO	Alkaloids	5.23
Adenine	C5H5N5	Alkaloids	0.81
calceolarioside A	C23H26O11	Phenylpropanoids	6.03
Chrysin	C15H10O4	Flavonoids	7.77
cirsimarin	C23H24O11	Flavonoids	8.09
Daidzein	C15H10O4	Flavonoids	10.16
Emodin	C15H10O5	Anthraquinones	7.16
Flavone base + 3O, 2MeO, O-Hex	C23H24O12	Flavonoids	7.05
Galanthamine HBr	C17H22BrNO	Miscellaneous	4.09
Ganoderic acid G	C30H44O8	Terpenoids	10.07
genipin 1-gentiobioside	C23H34O15	Terpenoids	3.63
Genistein	C15H10O5	Flavonoids	8.7
Glycitein	C16H12O5	Flavonoids	7.73
Hematoxylin	C16H14O6	Flavonoids	6.76
Hydroxygenkwanin	C16H12O6	Flavonoids	8.4
Iridin	C24H26O13	Flavonoids	7.22
Luteolin	C15H10O6	Flavonoids	7.14
Methyl 4-hydroxy-3-methoxycinnamate	C11H12O4	Phenolic acids	4.81
Nicotinic acid	C6H5NO2	Alkaloids	0.42
Periplogenin	C23H34O5	Terpenoids	12.76
Kaempferol	C15H10O6	Flavonoids	6.92
Kaempferol-3-glucoside	C21H20O11	Flavonoids	6.26
Vitamin D3	C27H44O	Terpenoids	12.08
Apigenin	C15H10O5	Flavonoids	10.92
alpha-Linolenic acid	C18H30O2	Fatty Acyls	11.94
Wogonin	C16H12O5	Flavonoids	26.83
Biochanin_A	C16H12O5	Flavonoids	14.23
LOVASTATIN	C24H36O5	Terpenoids	13.38
Kaempferol-3-O-glucuronoside	C21H18O12	Flavonoids	6.23
Kaempferide	C16H12O6	Flavonoids	10.88
Kaempferol-3-O-rutinoside	C27H30O15	Flavonoids	5.9
Isoquercitrin	C21H20O12	Flavonoids	5.68
Deoxyandrographolide	C20H30O4	Terpenoids	17.17
Naringin dihydrochalcone	C27H34O14	Flavonoids	6.95
Coumaric acid	C9H8O3	Phenylpropanoids	6.65
Etoposide	C29H32O13	Lignans	6.3

Acetyl-11-keto-beta-boswellic acid	C32H48O5	Terpenoids	16.5
Geniposide	C17H24O10	Iridoids	4.25
Betaine	C5H11NO2	Alkaloids	26.87
4-Guanidinobutyric acid	C5H11N3O2	Organic acids and derivatives	0.74
Fisetin	C15H10O6	Flavonoids	9.03
Hydroprotopine	C20H20NO5+	Alkaloids	6.98
Canthaxanthin (Euglenanone)	C40H52O2	Prenol lipids	17.97
Wogonoside	C22H20O11	Flavonoids	17.97
Poncirin	C28H34O14	Flavonoids	7.21
Acacetin	C16H12O5	Flavonoids	9.83
3,4-Dihydrocoumarin	C9H8O2	Terpenoids	1.34
Cyclo(leucylprolyl)	C11H18N2O2	Miscellaneous	5.04
Choline	C5H14NO	Organonitrogen compounds	3.95
N-Methyl-2-pyrrolidone	C5H9NO	Ketones	1.33
BIOTIN	C10H16N2O3	Alkaloids	26.84
Isoflavone base + 3O	C15H10O5	Flavonoids	17.13
PROLINE	C5H9NO2	Amino acid derivatives	0.7
L-Valine	C5H11NO2	Alkaloids	29.69
Isopropyl 4-Hydroxybenzoate	C10H12O3	Phenolic acids	4.25
4-Phenyl-3-buten-2-one	C10H10O	Benzene and substituted derivatives	8.87
Apigenin-6-C-glucoside-7-O-glucosic	C27H30O15	Flavonoids	6.5
LPC 16:0	C24H50NO7I	Lipids	13.65
Guanine	C5H5N5O	Alkaloids	0.81
AZELAIC ACID	C9H16O4	Fatty Acyls	6.94
Isoleucine	C6H13NO2	Amino acid derivatives	0.92
4-Aminophenol	C6H7NO	Benzene and substituted derivatives	0.81
Docosahexaenoic acid	C22H32O2	Fatty acids	15.62
Tanshinone IIA	C19H18O3	Diterpenoids	13.65
Anisic aldehyde	C8H8O2	Phenols	4.79
Oroxylin A-7-O-beta-D-glucuronide	C22H20O11	Flavonoids	17.12
Carveol	C10H16O	Prenol lipids	3.62
Bufalin	C24H34O4	Terpenoids	13.85
Maritimein	C21H20O11	Flavonoids	5.87
Tyrosine	C9H11NO3	Amino acid derivatives	0.82

Tectochrysin	C16H12O4	Flavonoids	8.47
Salicylic acid	C7H6O3	Phenols	2.66
Cryptotanshinone	C19H20O3	Diterpenoids	12.58
Scopoletin	C10H8O4	Coumarins and derivatives	5.34
Acetylcorynoline	C23H23NO6	Alkaloids	8.71
(5xi,9xi,10alpha)-Kaur-16-en-18-oic acid	C20H30O2	Terpenoids	13.22
loganic acid	C16H24O10	Organic acids and derivatives	4.34
Phenethylacetate	C10H12O2	Benzene and substituted derivatives	14.97
Isofraxidin	C11H10O5	Phenylpropanoids	6.82
Phenelzine	C8H12N2	Organic compound	5.46
Flavone base + 3O, O-HexA	C21H18O11	Flavonoids	5.9
7-Hydroxycoumarin	C9H6O3	Coumarins and derivatives	8.17
Jasminoside B	C16H26O8	Triterpenoids	3.08
Phthalic anhydride	C8H4O3	Miscellaneous	13.45
Linolenic acid ethyl ester	C20H34O2	Miscellaneous	13.85
Cinnamic acid	C9H8O2	Phenylpropanoids	3.63
Cimifugin	C16H18O6	Flavonoids	6.07
Isophorone	C9H14O	Organooxygen compounds	2.39
Arginine	C6H14N4O2	Amino acid derivatives	0.72
1-O-b-D-glucopyranosyl sinapate	C17H22O10	Phenylpropanoids	3.95
Clareolide	C16H26O2	Terpenoids	16.97
Ursolic acid	C30H48O3	Terpenoids	15.22
Adenosine	C10H13N5O4	Alkaloids	0.82
Vanillin	C8H8O3	Phenols	4.58
2,3-dihydroxypropyl hexadecanoate	C19H38O4	Miscellaneous	16.1
Baicalin	C21H18O11	Flavonoids	11.41
Tauroursodeoxycholic acid	C26H45NO6S	Terpenoids	7.82
Jatrorrhizine	C20H20NO4+	Alkaloids	8.1
Tyramine	C8H11NO	Alkaloids	14.12
3,5-Dicaffeoylquinic acid	C25H24O12	Phenylpropanoids	6.54
8-Methoxypsoralen	C12H8O4	Phenylpropanoids	4.84
Cearoin	C14H12O4	Phenols	6.96
5-Hydroxymethylfurfural	C6H6O3	Organooxygen compounds	1.45
neoeriocitrin	C27H32O15	Flavonoids	6.39

Cholic acid	C24H40O5	Terpenoids	10.07
Lutl-6-C-Glc	C21H20O11	Flavonoids	4.57
Apigenin-7-O-glucoside	C21H20O10	Flavonoids	8.15
Quebrachitol	C7H14O6	Miscellaneous	0.82
Zizyberanalic acid	C30H46O4	Terpenoids	12.12
Sinapoyl aldehyde	C11H12O4	Phenylpropanoids	4.23
Phenylalanine	C9H11NO2	Amino acid derivatives	1.33
20-Hydroxyecdysone 20,22-acetonide	C30H48O7	Terpenoids	14.61
Spirost-5-en-3-ol, (3beta,25R)-	C27H42O3	Terpenoids	11.73
Baicalin methyl ester	C22H20O11	Flavonoids	7.48
Nardosinone	C15H22O3	Terpenoids	5.99
7-Methoxycoumarin	C10H8O3	Coumarins and derivatives	1.56
Glabrolide	C30H44O4	Terpenoids	16.15
Sparteine	C15H26N2	Alkaloids	14.69
Histamine	C5H9N3	Alkaloids	24.18
Isoguanosine	C10H13N5O5	Alkaloids	0.82
Vicenin II	C27H30O15	Flavonoids	6.24
Naringenin	C15H12O5	Flavonoids	7.41
7-Hydroxy-4-methylcoumarin	C10H8O3	Phenylpropanoids	6.03
Thymol	C10H14O	Phenols	6.77
Pristimerin	C30H40O4	Terpenoids	12.07
Corylin	C20H16O4	Flavonoids	0.62
D-Pantothenic acid	C9H17NO5	Organic oxygen compounds	1.62
Boldine	C19H21NO4	Alkaloids	5.97
Perillene	C10H14O	Terpenoids	27.77
Sakuranetin	C16H14O5	Flavonoids	7.59
Daidzein-8-C-glucoside	C21H20O9	Flavonoids	6.47
Erucamide	C22H43NO	Amides	18.45
Camphor	C10H16O	Terpenoids	5.9
Sciadopitysin	C33H24O10	Flavonoids	13.14
Sarracenin	C11H14O5	Iridoids	4.26
Lysionotin	C18H16O7	Flavonoids	9.98
Sinapic acid	C11H12O5	Phenylpropanoids	4.02
7-Methoxy-4-methylcoumarin	C11H10O3	Coumarins and derivatives	6.75
(-)-Syringaresinol di-O-glucoside	C34H46O18	Phenylpropanoids	5.47
Abietic acid	C20H30O2	Terpenoids	15
Dehydrodiisoeugenol	C20H22O4	Lignans	12.27
Acetophenone	C8H8O	Phenols	3.63
skullcapflavone II	C19H18O8	Flavonoids	16.07

Artemisinic acid	C15H22O2	Terpenoids	5.66
Ascorbic acid	C6H8O6	Dihydrofurans	4.23
(+)-Corynoline	C21H21NO5	Alkaloids	8.31
gardenoside	C17H24O11	Sesquiterpenoids	2.03
Dexamethasone	C22H29FO5	Terpenoids	0.2
Visnagin	C13H10O4	Flavonoids	10.75
Chlorogenic acid	C16H18O9	Phenylpropanoids	3.16
Casticin	C19H18O8	Flavonoids	15.85
Testosterone	C19H28O2	Terpenoids	11.73
Isoschaftoside	C26H28O14	Flavonoids	5.05
Rosmarinic acid	C18H16O8	Phenylpropanoids	9.48
Methyl vanillate	C9H10O4	Phenolic acids	27.73
Veratramine	C27H39NO2	Alkaloids	11.06
Androstane-3,17-diol	C19H32O2	Terpenoids	14.56
Maltol	C6H6O3	Flavonoids	0.81
(-)-12-hydroxyjasmonic acid	C12H18O4	Jasmonic acid	13.56
Wilforlide A	C30H46O3	Terpenoids	13.57
7,8-dihydromethysticin	C15H16O5	Miscellaneous	8.82
Isosteviol	C20H30O3	Terpenoids	11.51
Flavone base + 3O, O-HexA-HexA	C27H26O17	Flavonoids	6.41
Pelargonidin-3-O-glucoside	C21H21O10	Flavonoids	7.55
Biochanin-7-O-glucoside	C22H22O10	Flavonoids	5.64
gamma-Linolenic acid	C18H30O2	Organic acids and derivatives	13.01
beta-Elemonic acid	C30H46O3	Terpenoids	14.19
Coniferyl aldehyde	C10H10O3	Phenylpropanoids	2.33
khelloside	C19H20O10	Ketones	4.02
Laetanine	C18H19NO4	Alkaloids	7.25
5-O-Demethylnobiletin	C20H20O8	Flavonoids	11.57
Kanzonol C	C25H28O4	Phenylpropanoids	4.48
Liquidambaric acid	C30H46O3	Terpenoids	12.63
plumieride	C21H26O12	Iridoids	6.65
β-Gentiobiose	C12H22O11	Miscellaneous	0.92
Hydroquinone	C6H6O2	Phenolic acids	5.36
Shanzhiside methyl ester	C17H26O11	Terpenoids	1.16
Silychristin	C25H22O10	Flavonoids	11.28
Acetylcarnitine	C9H18NO4	Miscellaneous	0.75
Gal(alpha1-6)Glc(alpha1-2beta)Fruf	C18H32O16	Miscellaneous	0.64
Pilocarpine	C11H16N2O2	Alkaloids	0.85
Scoparone	C11H10O4	Coumarins and derivatives	8.41
Syringetin-3-O-glucoside	C23H24O13	Flavonoids	5.14
Lithospermic acid	C27H22O12	Phenylpropanoids	9.46

12-oxo-phytodienoic acid	C18H28O3	Organic acids and derivatives	10.87
2-Hydroxycinnamic acid, predominantly trans	C9H8O3	Phenylpropanoids	0.82
Glycocholic acid	C26H43NO6	Terpenoids	7.65
sclareol	C20H36O2	Terpenoids	15.56
Ligustilide	C12H14O2	Dihydrofurans	14.34
Oxypeucedanin hydrate	C16H16O6	Coumarins and derivatives	7.6
Ginkgolic acid (C13:0)	C20H32O3	Organic acids and derivatives	13.98
Pesticide3_Propoxur_C11H15NO3_Baygon	C11H15NO3	Miscellaneous	3.17
asperuloside	C18H22O11	Iridoids	0.63
Kainic acid	C10H15NO4	Organic acids and derivatives	25.66
Polygalic acid	C29H44O6	Organic acids and derivatives	15.7
Gardenin B	C19H18O7	Flavonoids	8.56
Undulatoside A	C16H18O9	Phenols	3.64
4-Nitrophenol	C6H5NO3	Phenols	0.82
DL-Coniine	C8H17N	Alkaloids	26.71
Glutamylphenylalanine	C14H18N2O5	Carboxylic acids and	2.35
Bruceine A	C26H34O11	Terpenoids	7.08
Cianidanol	C15H14O6	Phenols	5.05
Isopeonol	C9H10O3	Phenols	4.26
Harpagoside	C24H30O11	Phenylpropanoids	6.65
Steviol-19-O-glucoside	C26H40O8	Terpenoids	8.65
p-Coumaric acid	C9H8O3	Phenylpropanoids	6.65
Tazettine	C18H21NO5	Alkaloids	4.94
Protopine	C20H19NO5	Alkaloids	8.2
Harmol	C12H10N2O	Alkaloids	6.34
1,7-Diphenylhept-4-en-21-one	C19H20O	Ester	6.76
Hecogenin	C27H42O4	Terpenoids	11.09
Hydrastine hydrochloride5936-28-7	C21H22ClNO	Alkaloids	8.97
Crocetin	C20H24O4	Miscellaneous	10.25
Dihydrocapsaicin	C18H29NO3	Alkaloids	9.02
oleuropein	C25H32O13	Terpene glycosides	6.03
Dihydrosanguinarine	C20H15NO4	Alkaloids	26.71
Peimisine	C27H41NO3	Alkaloids	16.77
monolinolein	C21H38O4	Phospholipid	15.25

Silibinin	$C_{25}H_{22}O_{10}$	Flavonoids	12.63
monoolein	$C_{21}H_{40}O_4$	Ester	16.36
Artemetin	$C_{20}H_{20}O_8$	Flavonoids	10.93
Stachydrine	$C_7H_{13}NO_2$	Alkaloids	0.75
(+)-Magnoflorine	$C_{20}H_{24}NO_4^+$	Alkaloids	4.78
swertiamarin	$C_{16}H_{22}O_{10}$	Iridoids	5.13
Verbascoside	$C_{29}H_{36}O_{15}$	Phenylpropanoids	6
Norboldine	$C_{18}H_{19}NO_4$	Alkaloids	6.3
Daidzin	$C_{21}H_{20}O_9$	Flavonoids	4.89
