# Supplementary material for: Network pharmacology analysis reveals neuroprotective effects of the Qin-Zhi-Zhu-Dan Formula in Alzheimer’s disease
Source: Front Neurosci. 2022 Oct 20;16:943400. doi: 10.3389/fnins.2022.943400 (PMC9632440; doi:10.3389/fnins.2022.943400)
Supplement: Supplementary file 4 [file Table_4.docx]

Supplementary Material

| Table S4. 15 potential compounds of QZZD and their ADME/T parmeters | | | | | | |
| --- | --- | --- | --- | --- | --- | --- |
| PubChem CID | Molecule Name | Structure | MW | AlogP | Hdon | Hacc |
| 64982 | baicalin | 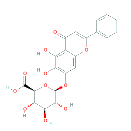 | 446.364 | 1.1 | 6 | 11 |
| 107848 | geniposide | 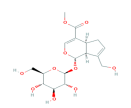 | 388.369 | -2.3 | 5 | 10 |
| 92805 | hyocholic acid | 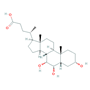 | 408.579 | 3.9 | 4 | 5 |
| 5283820 | hyodeoxycholic acid | 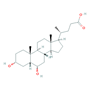 | 392.58 | 4.9 | 3 | 4 |
| 10133 | Chenodeoxycholic acid | 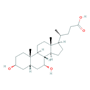 | 392.58 | 4.9 | 3 | 4 |
| 9903 | Lithocholic acid | 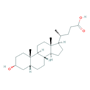 | 376.581 | 6.3 | 2 | 3 |
| 1123 | Taurine | 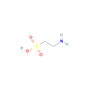 | 125.142 | -4.1 | 2 | 4 |
| 750 | Glycine | 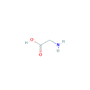 | 75.067 | -3.2 | 2 | 3 |
| 11954195 | Taurohyocholic acid | 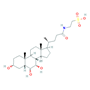 | 515.706 | 2.6 | 5 | 7 |
| 119046 | Taurohyodeoxycholic acid | 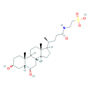 | 499.707 | 3.6 | 4 | 6 |
| 387316 | Taurochenodeoxycholic acid | 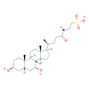 | 499.707 | 3.6 | 4 | 6 |
| 439763 | Taurolithocholic acid | 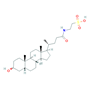 | 483.708 | 4.9 | 3 | 5 |
| 71361462 | Glycohyocholic acid | 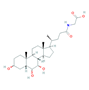 | 465.631 | 3.3 | 5 | 6 |
| 114611 | Glycohyodeoxycholic acid | 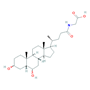 | 449.632 | 4.3 | 4 | 5 |
| 12544 | Glycochenodeoxycholic acid | 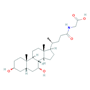 | 449.632 | 4.3 | 4 | 5 |

Note: We determine the specific measurement principles of ADME/T according to the Lipinski‘s Rule (i.e. *MW<500*, *AlogP<5*, *Hdon<5*, and *Hacc<10*).
